# Zonula occludens-1 expression is reduced in nasal epithelial cells of allergic rhinitis patients

**DOI:** 10.7717/peerj.13314

**Published:** 2022-04-22

**Authors:** Che Othman Siti Sarah, Siti Muhamad Nur Husna, Norasnieda Md. Shukri, Kah Keng Wong, Noor Suryani Mohd Ashari

**Affiliations:** 1 Department of Immunology, School of Medical Sciences, Universiti Sains Malaysia, Kubang Kerian, Kelantan, Malaysia; 2 Department of Otorhinolaryngology, Head and Neck Surgery, School of Medical Sciences, Hospital Universiti Sains Malaysia, Universiti Sains Malaysia, Kubang Kerian, Kelantan, Malaysia

**Keywords:** Allergic rhinitis, Zonula occludens, Histone deacetylase, House dust mites, *ZO-1*, *ZO-2*, *ZO-3*, *HDAC1*, *HDAC2*

## Abstract

Allergic rhinitis (AR) is a common allergic disease characterized by disruption of nasal epithelial barrier. In this study, we investigated the mRNA expression of zonula occludens-1 (*ZO-1*), *ZO-2* and *ZO-3* and histone deacetylase 1 (*HDAC1*) and *HDAC2* in AR patients compared to healthy controls. RNA samples were extracted from nasal epithelial cells of house dust mites (HDMs)-sensitized AR patients and healthy controls (*n* = 28 in each group). The RNAs were reverse transcribed into cDNAs for measurement of *ZO-1*, *ZO-2*, *ZO-3*, *HDAC1* and *HDAC2* expression levels by quantitative PCR. The mRNA expression of *ZO-1* was significantly decreased in AR patients compared to healthy controls (*p* = 0.010). No significant difference was observed in the expression levels of *ZO-2*, *ZO-3*, *HDAC1* and *HDAC2* in AR patients compared to healthy controls. We found significant associations of higher *HDAC2* levels in AR patients with lower frequency of changing bedsheet (*p* = 0.043) and with AR patients sensitized to *Dermatophagoides farinae* (*p* = 0.041). Higher expression of *ZO-2* was observed in AR patients who had pets (*p* = 0.007). In conclusion, our data indicated that *ZO-1* expression was lower in AR patients contributing to decreased integrity of nasal epithelial barrier integrity, and HDAC2 may be involved in the pathogenesis of the disease.

## Introduction

Allergic rhinitis (AR) is an IgE-mediated inflammation disorder of the nasal mucosa caused by infiltration of allergens (Varshney & Varshney, 2015). This leads to imbalanced immunological reaction resulting in clinical manifestations of the disease including sneezing, nasal obstruction, nasal pruritus, rhinorrhoea or conjunctivitis (Sin & Togias, 2011; Wheatley & Togias, 2015; Brozek et al., 2017; Siti Sarah et al., 2020). AR is a common disease affecting 10% to 30% of adults and up to 40% of children (Mims, 2014). AR is classified into four groups based on the Allergic Rhinitis and its Impact on Asthma (ARIA) guidelines, which are mild intermittent, mild persistent, moderate-severe intermittent and moderate-severe persistent (Klimek et al., 2019). Exposure to allergens such as grass, tree pollen, house dust mites (HDMs), animal dander, foods, insect venoms and medicines induce the production of IgE that subsequently triggers allergic reaction (Galli, Tsai & Piliponsky, 2008; Ong & Chew, 2009; Yang et al., 2018; Fassio & Guagnini, 2018; Wang et al., 2018b). HDMs and pollen allergens have protease activity that contribute to the disruption of nasal epithelial barrier (Wan et al., 1999; Roche et al., 2000; Okano, 2009; Matsumura, 2012). The decreased of epithelial barrier integrity will result in an increased in allergen invasion and contribute to the pathogenesis of AR (Steelant et al., 2016; Wang et al., 2020a; Wang et al., 2020b). Thus, the decrease of ZO-1 level is responsible for symptoms of AR. Pollen-derived proteases have been shown to degrade occludin, and facilitate the allergen delivery through epithelia by disrupting the epithelial tight junction (TJ) components (Matsumura, 2012). *Dermatophagoides pteronyssinus* (*D. pteronyssinus*) delivers cysteine proteases that increases the epithelial permeability by disrupting claudin-1, occludin and ZO-1 molecules (Baker et al., 2003; Matsumura, 2012; Henriquez et al., 2013).

Nasal airways are exposed to the environmental allergens caused by the breakdown of the nasal epithelial barrier that blocks the passage to allergens. The gap between the epithelial cells are closed by TJ molecules and form a complex structure to protect the body from infiltration of external allergens (Beutel et al., 2019). TJ molecules consist of transmembrane proteins (*e.g.,* occludin, claudins and tricellulin) and junctional adhesion molecules (Steelant et al., 2016; Heinemann & Schuetz, 2019). The scaffold adaptor protein zonula occludens (ZO) forms a TJ assembly attached to actin cytoskeleton and TJ molecules (Beutel et al., 2019). ZO proteins are expressed in epithelial cells throughout the airway where they remain localized to the cell borders (Smith, Koval & Levy, 2020).

ZO proteins are from the MAGUK family members including ZO-1, ZO-2 and ZO-3, and they are crucial in maintaining the epithelial barrier integrity (Itoh et al., 1999; Chelakkot, Ghim & Ryu, 2018). Decreased expression of ZO-1 occur in the nasal epithelium cells of AR patients (Lee et al., 2016). ZO-1 and ZO-2 expression were also reduced in patients with chronic rhinosinusitis (CRS) without nasal polyps (Soyka et al., 2012). Defects of these ZO proteins may contribute to the opening of the gap between epithelium cells and the dysfunction of nasal epithelial barrier.

Histone deacetylase (HDAC) is a group of enzymes that can repress the transcription of gene through deacetylation of histone molecules. HDAC acts by removing an acetyl group from lysine on histones (Jiang et al., 2015). HDACs regulate expression of genes involved in multiple cellular activities including inflammatory responses leading to allergies (Bhavsar, Ahmad & Adcock, 2008; Barnes, 2013). A study using a mouse model with AR fed with HDAC inhibitor (HDACi) sodium butyrate (NaB), showed positive effects to treat AR by decreasing the expression of HDAC1 and HDAC8 (Wang et al., 2020a; Wang et al., 2020b). Higher expression of HDAC1 can cause further defects of ZOs expression (Lei et al., 2010; Zhou et al., 2015). HDAC1 has been found to suppress Trek1 and the exposure to the signature T helper 2 (Th2) cytokine, interleukin (IL)-4, upregulated the expression of HDAC1 resulting in significantly suppressed expression of Trek1 in the nasal mucosa (Jiang et al., 2015). These findings highlighted the importance of HDAC1 as a central player in the mechanism regulating the outcome of epithelial barrier function, and HDAC1 is a potential target for inhibition to ameliorate nasal epithelial barrier dysfunction for better outcome in AR. HDAC1 inhibition had been shown to increase the TJ expression while decreasing epithelial barrier integrity defects (Wawrzyniak et al., 2017). *ZO-1* mRNA expression level was found to be lower in AML-12 murine hepatocyte cells with the presence of overexpressed HDAC1 (Lei et al., 2010). HDAC2 inhibited claudin-1 expression in malignant epithelial cells (Krishnan et al., 2010) and HDACs are known to suppress TJs expression in other disease contexts (Sin & Togias, 2011; Brozek et al., 2017). Human lens epithelial cells treated with Trichostatin-A (TSA), a HDACi decreased HDAC2 and increased the mRNA expression of *ZO-1* (Ganatra et al., 2018). In addition, treatment of NCM460 cells with CAY 10683, a HDAC2 inhibitor increased ZO-1 mRNA and protein levels (Wang et al., 2018a).

Past studies have focused on the association of ZO-1 and HDAC1 gene expressions in moderate/severe HDM-induced AR patients; these HDMs include *Dermatophagoides farinae* (*D. farinae*), *D. pteronyssinus* and *Blomia tropicalis* (*B. tropicalis*), which are the most common aeroallergens affecting AR population in Malaysia (Mohd Ashari et al., 2014; Lim et al., 2015; Azid et al., 2019; Sani et al., 2019; Nur Husna et al., 2021). There is lack of literature in association of ZO-2, ZO-3 and HDAC2 in moderate /severe HDM-induced AR patients. Moreover, data associating ZO and HDAC to environmental or lifestyle factors such as having pets remain scarce.

Our gene expression studies and their associations with clinico-demographical and environmental parameters in AR patients are divided into two parts. The first part of this study focused on ZO proteins and HDACs, while the second part focused on occludin, claudins and desmogleins. In this study, we aimed to investigate the mRNA expression of ZOs (*ZO-1*, *ZO-2* and *ZO-3*) and HDACs (*HDAC1* and *HDAC2*) in AR patients compared to healthy controls in subjects recruited from Hospital Universiti Sains Malaysia, Kelantan, Malaysia. The associations of the expression levels of ZOs and HDACs with the clinico-demographical parameters of AR patients and healthy controls, as well as patients’ lifestyle were measured.

## Materials & Methods

### Subjects recruitment

Participants were recruited from Otorhinolaryngology-Head & Neck Surgery (ORL-HNS) clinic, Universiti Sains Malaysia (USM), Kubang Kerian, Kelantan, Malaysia. A total of 28 moderate-severe persistent and intermittent AR patients were recruited, and classification of AR patients was based on the ARIA guidelines (Klimek et al., 2019). Healthy control subjects (*n* = 28) were volunteers and they were staffs or students within the Health Campus, USM. Only adults of 18 years and above were chosen to participate in this study, and they were legally competent to fill in the written consent. The Human Research Ethics Committee of Universiti Sains Malaysia (JEPeM) authorized the study’s protocols (approved ethics code: USM/JEPeM/18060273).

Sample size was calculated according to the difference of means between two independent groups using the software Power and Sample Size (PS) version 3.1.9.3 (Vanderbilt University, Nashville, Tennessee, USA; 2011) based on previous qPCR studies of nasal mucosa tissue samples (Kamekura et al., 2009; Steelant et al., 2016; Steelant et al., 2018; Zhao et al., 2018). A two-tailed hypothesis with 0.05 α-error probability, 0.8 power (1-β error probability), 0.75 effect size, one allocation ratio (N2/N1) and 5% dropout rate. This resulted in a total sample size of 56 subjects divided evenly between AR patients and healthy controls groups (*n* = 28 in each group).

During the recruitment process, all participants were given detailed information about the study. The written consents were obtained from the participants for the investigators to proceed with the recruitment, performing SPT, taking participants’ nasal epithelial samples via nasal brushing for experiments and publish the results. All samples were labelled anonymously, and handling of all data was conducted anonymously where none of the participant’s private information such as name was disclosed. All experimental procedures were carried out in accordance with the institutional guidelines and regulations.

### Allergen Skin Prick Test (SPT)

Allergen SPT was performed to the subjects prior to collection of nasal epithelial cells samples. Saline was used as the negative control, histamine as the positive control and allergen extracts were applied to the skin. Allergen extracts consisted of three types of HDMs *i.e.,* *D. pteronyssinus*, *D. farinae* and *B. tropicalis*. Sterile lancets (ALK-Abelló A/S, Hørsholm, Denmark) were used to prick the skin gently through the drops of allergen. The reactions were observed after 15 to 30 min. The formation of wheal indicated the presence of antibodies against the allergen. Patients with wheal size of  ≥4 mm for HDM allergens and not for saline were considered positive for sensitization and they were recruited to participate in this study. Healthy controls were recruited if they did not show any reaction towards all the allergen extracts.

### Collection of Nasal Epithelial Cells (NECs)

Cytology brush was used to collect nasal epithelial cells by brushing softly the surface of nasal inferior turbinate. Inferior turbinate tissue biopsy is an alternative method for the collection of nasal epithelium and detection of allergen-specific IgE, however tissue biopsy methodology is invasive and it has recently been shown that nasal mucosal brushing is comparable with inferior turbinate tissue biopsy for the diagnosis of local AR (Hamizan et al., 2019). In addition, multiple independent studies have adopted nasal brushing method for the collection of nasal epithelium for gene expression analysis in AR patients (Giovannini-Chami et al., 2012; Imoto et al., 2013). The brush was wet with sterile isotonic solution before using on each nostril, for patients and control subjects. The brush was rubbed a few times rapidly against the medial and superior side of the inferior nasal meatus. The brush was immediately placed into a centrifuge tube filled with 350 µl RLT buffer solution (QIAGEN, Hilden, Germany) to preserve the RNA quality. The tube was kept in −80° before RNA extraction.

### RNA extraction

Total RNA was extracted using RNeasy^®^ Mini Kit (QIAGEN). Cells from nasal brushings in RLT buffer were pelleted by centrifugation (8,300 rpm, 5 min). One volume of 70% ethanol was added to the lysate and transferred to an RNeasy Mini spin column placed in a 2 ml collection tube. After the centrifugation, buffer RW1 was added to the column. The extraction was further continued with DNA digestion by adding DNase I stock solution mixed with Buffer RDD from RNase-Free DNase Set. The RW1 washing buffer was added to the column and ethanol was added again to increase the purity of the RNA yield. To improve the quality of the RNAs, two times of RPE washing buffer were added to the column. After centrifugation, the spin column was transferred to new collection tube and then spun once more to remove excess solution. Lastly, RNeasy spin column was placed into new collection tube and 40 µl RNase-free water was added to elute RNA. RNA quantity and quality were measured using Epoch™ Microplate spectrophotometer (Epoch™, Biotek, USA).

### cDNA synthesis

RNA samples were reverse-transcribed to cDNA using iScriptTM Reverse Transcription Supermix for RT-qPCR (Bio-Rad, Philadelphia, PA, USA). The RNA samples were synthesized to cDNA using the thermocycler GeneAmp® PCR System 9700 (Applied Biosystems, Waltham, USA). The PCR condition was set up at 25 °C for 5 min at priming, 46 °C for 20 min at reverse transcription and RT inactivation for 1 min at 95 °C for 1 cycle. The synthesized cDNA samples were measured using EpochTM Microplate spectrophotometer.

### Quantitative PCR (qPCR)

The cDNA samples were diluted 1:5 as template for qPCR conducted using the iTaQ Universal SYBR Green Super Mix (Bio-Rad, Philadelphia, PA, USA) and designed primers (*ZO-1*, *ZO-2*, *ZO-3*, *HDAC1* and *HDAC2*) using Mx3005p qPCR thermal cycler (Agilent Technologies, Santa Clara, CA, USA). The primers (Integrated DNA Technologies, Singapore) were designed using NCBI Primer-BLAST (Table 1). Each reaction was performed in triplicate with 1 cycle of DNA denaturation step at 95 °C for 25 s, 40 cycles of amplification at 95 °C for 5 min, and extension step at 60 °C for 25 s. The results were calculated using the 2^−ΔΔCt^ method and normalized to the control gene *GAPDH*.

**Table 1:** List of SYBR green primers used for qPCR.

**Target gene**	**Accession number**	**Forward primer (5′-3′)**	**Reverse primer (5′-3′)**	**Amplicon size (bp)**	**Primer spans exon junction**
***ZO-1***	NM_003257.4	TAAAGAGAAAGGTGAAACACTGCTG	ATCACAGTGTGGTAAGCGCA	100	Yes-Forward
***ZO-2***	NM_004817.4	GCTGCTCCAAGAAAATGACAGA	GGGGCCTCTTGACCACAATA	128	Yes-Forward
***ZO-3***	NM_001267560.2	GAGGAGAGACAGCGAAGAGTT	GTGTCGTTCAGTGACAGGTTC	162	Yes-Forward
***HDAC1***	NM_004964.3	CTACGACGGGGATGTTGGAA	CAGCATTGGCTTTGTGAGGG	143	Yes-Forward
***HDAC2***	NM_001527.4	TGCTACTACTACGACGGTGA	TGTCATTTCTTCGGCAGTGG	162	Yes-Forward
***GAPDH***	NM_002046.7	TCGGAGTCAACGGATTTGGT	TTCCCGTTCTCAGCCTTGAC	181	Yes-Forward

### Statistical analysis

All results were analyzed using Mann–Whitney U test in GraphPad Prism v6 (GraphPad Software, La Jolla, CA, USA). Shapiro–Wilk normality test was used to determine whether the data was normally distributed. Associations of the gene expression (median cut-off) with clinico-demographical and environmental parameters were examined using the *χ*2-test or Fisher’s exact test as appropriate. Median is a better measure of central tendency than mean as it is less affected by the influence of skewed data and outliers, hence median cut-off was chosen to analyze the associations between the gene expression and the clinico-demographical and environmental parameters. For all analysis, two-tailed *p*-values <0.05 were considered as statistically significant.

## Results

### Characteristics of the recruited subjects

The demographic data of the subjects are shown in Table 2. A total of 56 subjects (28 healthy controls and 28 moderate-severe AR patients) were enrolled in this study. Of the 28 healthy controls, nine were male (32.1%) and 19 were female (67.9%). The number of participants with persistent AR was higher than intermittent AR [*n* = 21 (75%) versus *n* = 7 (25%)]. The most commonly reported comorbidities by moderate-severe AR patients were sinusitis (*n* = 26; 92.9%), and conjunctivitis (*n* = 21; 75%). Other comorbidities include pharyngitis (*n* = 15; 53.6%), asthma (*n* = 11; 39.3%), otitis media (*n* = 5; 17.9%) and lymphoid hypertrophy or obstructive sleep apnea (*n* = 2; 7.14%). The number of participants exposed to secondhand cigarette smoke was higher in moderate-severe AR patients (*n* = 19; 67.9%) than in healthy controls (*n* = 13; 49.4%). There was no difference in home location (urban or rural) or living in an industrial area between healthy controls and moderate-severe AR patients.

**Table 2:** Characteristics of the healthy controls and HDM-sensitized AR patients.

**Characteristics**	**Healthy controls** **(*n* = 28)**	**Moderate-severe AR** **(*n* = 28)**
**Mean age (years)**±**SD**	30.82 ± 7.9	30.07 ± 8.9
**Gender**		
Male	9 (32.1)	9 (32.1)
Female	19 (67.9)	19 (67.9)
**Mean BMI (kg/m**^**2**^**)**±**SD**	24.42 ± 4.8	26.92 ± 4.9
**Immediate family history of allergy**	NA	23 (82.1)
**Classification**		
Intermittent	NA	7 (25.0)
Persistent	NA	21 (75.0)
**Comorbidities**		
Conjunctivitis	NA	21 (75.0)
Pharyngitis	NA	15 (53.6)
Sinusitis	NA	26 (92.9)
Asthma	NA	11 (39.3)
Otitis media	NA	5 (17.9)
Lymphoid hypertrophy/obstructive sleep apnea	NA	2 (7.14)
Speech impairment	NA	NA
**Exposure to secondhand smoke**	13 (46.4)	19 (67.9)
**Home location**		
Rural	14 (50.0)	13 (46.4)
Urban	14 (50.0)	15 (53.6)
**Living in an industrial area**	3 (10.7)	1 (3.6)
**Having pet**	17 (60.7)	16 (57.1)
**Frequency of changing bedsheet**		
Weekly	13 (46.4)	12 (42.9)
2-Monthly	2 (7.2)	4 (14.2)
Monthly	13 (46.4)	12 (42.9)
**Frequency of performing housekeeping**		
Daily	13 (46.4)	10 (35.7)
Alternate day	NA	7 (25.0)
Weekly	14 (50.0)	10 (35.7)
Monthly	1 (3.6)	1 (3.6)

**Notes.**

Abbreviations AR allergic rhinitis NA not applicable BMI body mass index SD standard deviation

### Expression profile of ZOs and HDACs in AR patients compared to healthy controls

**Figure 1:**
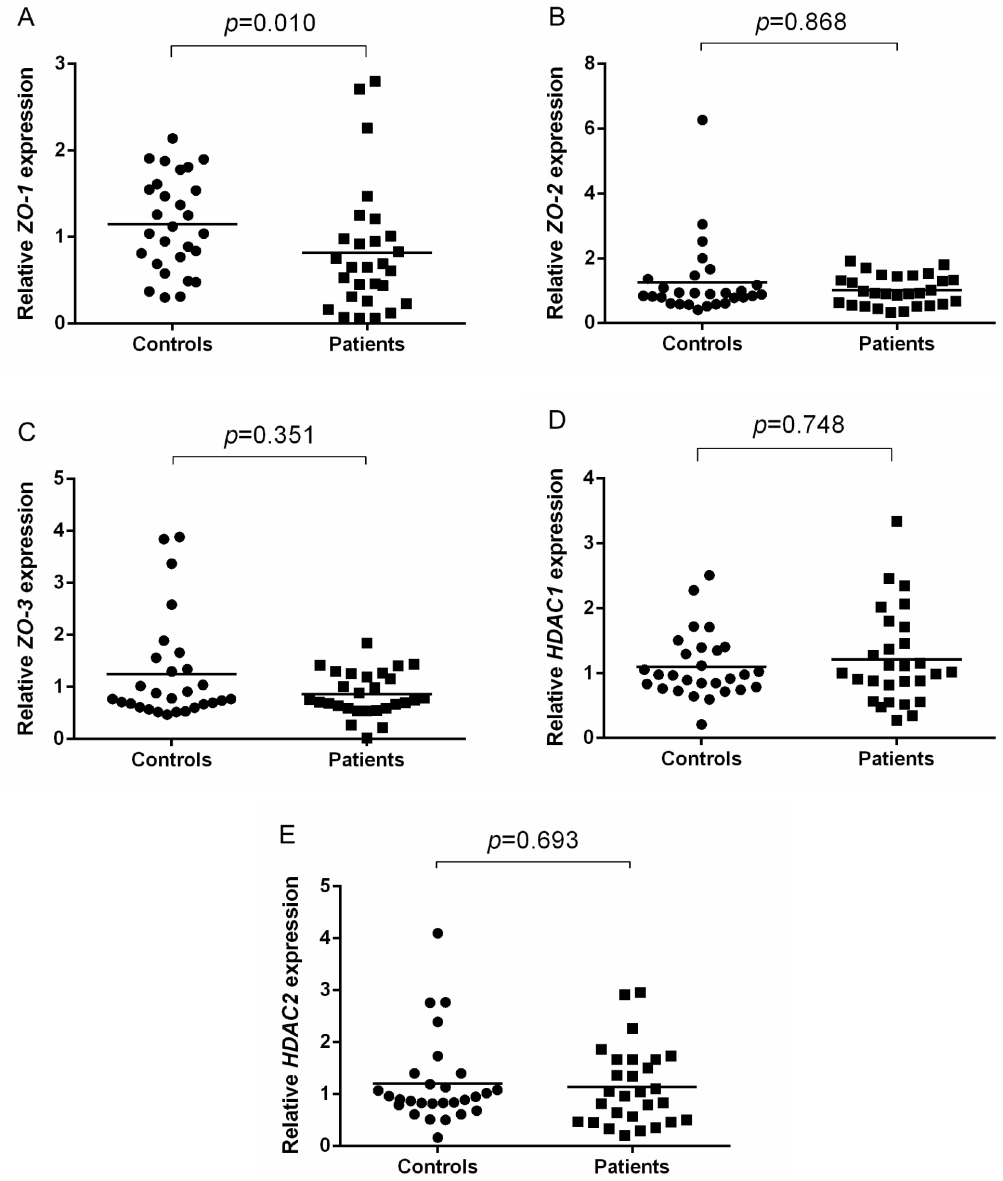
Relative *ZOs* and *HDACs* expression of AR patients and healthy controls. (A) Relative *ZO-1* expression of AR patients and healthy controls. (B) Relative *ZO-2* expression of AR patients and healthy controls. (C) Relative *ZO-3* expression of AR patients and healthy controls. (D) Relative *HDAC1* expression of AR patients and healthy controls. (E) Relative *HDAC2* expression of AR patients and healthy controls.

To address potential role of TJ molecules in maintaining the epithelial barrier integrity, *ZO-1*, *ZO-2, ZO-3*, *HDAC1* and *HDAC2* mRNA expression were measured in NECs of AR patients and healthy controls. Shapiro–Wilk normality test showed that the data were not normally distributed. Thus, Mann–Whitney U test was used. *ZO-1* expression was significantly lower in AR patients (*p* = 0.010) compared to controls (Fig. 1A). No difference was found in the expression of *ZO-2* (*p* = 0.868) and *ZO-3* (*p* = 0.351) in AR patients compared to controls (Figs. 1B and 1C).

In terms of HDACs expression, no significant difference in the expression of *HDAC1* (*p* = 0.748) and *HDAC2* (*p* = 0.693) in AR patients compared to healthy controls (Figs. 1D and 1E).

### Association of ZOs and HDACs expression with demographical and clinical parameters of AR patients and healthy controls

The expression of each gene (median cut-off) was examined in terms of their association with demographical parameters of AR patients and healthy controls. Higher *HDAC2* expression was significantly associated with lower frequency of changing bedsheet (*p* = 0.043) (Table 3) and sensitization to HDM (*D. farinae*) (*n* = 14/23; *p* = 0.041) (Table 4) in patients with AR. There was no significant differences between gender, age, BMI, home location, having pet, frequency of changing bedsheet and doing housekeeping with each gene expression (*ZO-1*, *ZO-2*, *ZO-3*, *HDAC1* and *HDAC2*) (Table 3). Family history of allergies, classification of persistent and intermittent AR patients, comorbidities (conjunctivitis, pharyngitis, sinusitis, asthma, otitis media, obstructive sleep apnea) and sensitivity to *D. pteronyssinus* and *B. tropicalis* were also not showed significant differences with each gene expression (*ZO-1*, *ZO-2*, *ZO-3*, *HDAC1* and *HDAC2*) of AR patients (Table 4).

In healthy controls, *ZO-2* expression was significantly higher in those with pets at home (*n* = 12/17; *p* = 0.007) (Table 5). Other parameters (gender, age, BMI, exposure to cigarette smoke, home location, frequency of housekeeping and changing bedding) had no significant differences with the expression of each gene’s expression (*ZO-1*, *ZO-2*, *ZO-3*, *HDAC1* and *HDAC2*) (Table 5).

**Table 3:** Association of each gene’s expression (*ZO-1*, *ZO-2*, *ZO-3*, *HDAC1* and *HDAC2*) with demographical parameters of AR patients (*n* = 28). *p* < 0.05 shown in bold.

**Characteristics**	***ZO-1*** **expression**			***ZO-2*** **expression**			***ZO-3*** **expression**			***HDAC1*** **expression**			***HDAC2*** **expression**		
	**<Median,** **n (%)**	**≥Median,** **n (%)**	***p*** **-value**	**<Median,** **n (%)**	**≥Median,** **n (%)**	***p*** **-value**	**<Median,** **n (%)**	**≥Median,** **n (%)**	***p*** **-value**	**<Median,** **n (%)**	**≥Median,** **n (%)**	***p*** **-value**	**<Median,** **n (%)**	**≥Median,** **n (%)**	***p*** **-value**
**Gender**															
**Male**	6 (21)	3 (11)	0.228 (F)	5 (18)	4 (14)	0.689 (F)	3 (11)	6 (21)	0.420 (F)	5 (18)	4 (14)	1.000 (F)	3 (11)	6 (21)	0.420 (F)
**Female**	7 (25)	12 (43)		8 (29)	11 (39)		11 (39)	8 (29)		9 (32)	10 (36)		11 (39)	8 (29)	
**Age (years)**															
**<28**	5 (18)	9 (32)	0.256	7 (25)	7 (25)	0.704	5 (18)	9 (32)	0.131	5 (18)	9 (32)	0.131	5 (18)	9 (32)	0.131
**≥28**	8 (29)	6 (21)		6 (21)	8 (29)		9 (32)	5 (18)		9 (32)	5 (18)		9 (32)	5 (18)	
**BMI**															
**<23.31**	7 (25)	7 (25)	0.704	5 (18)	9 (32)	0.256	5 (18)	9 (32)	0.131	6 (21)	8 (29)	0.450	6 (21)	8 (29)	0.450
**≥23.31**	6 (21)	8 (29)		8 (29)	6 (21)		9 (32)	5 (18)		8 (29)	6 (21)		8 (29)	6 (21)	
**Exposure to secondhand smoke**															
**No**	3 (11)	6 (21)	0.435 (F)	6 (21)	3 (11)	0.228 (F)	6 (21)	3 (11)	0.420 (F)	6 (21)	3 (11)	0.420 (F)	6 (21)	3 (11)	0.420 (F)
**Yes**	10 (36)	9 (32)		7 (25)	12 (43)		8 (29)	11 (39)		8 (29)	11 (39)		8 (29)	11 (39)	
**Home Location**															
**Rural**	7 (25)	6 (21)	0.464	8 (29)	5 (18)	0.136	8 (29)	5 (18)	0.256	6 (21)	7 (25)	0.704	7 (25)	6 (21)	0.704
**Urban**	6 (21)	9 (32)		5 (18)	10 (36)		6 (21)	9 (32)		8 (29)	7 (25)		7 (25)	8 (29)	
**Living in an industrial area**															
**No**	12	15	0.464 (F)	13	14	1.000 (F)	14	13	1.000 (F)	14	13	1.000 (F)	14	13	1.000 (F)
**Yes**	1	NIL		NIL	1		NIL	1		NIL	1		NIL	1	
**Having pet**															
**No**	6 (21)	6 (21)	0.742	7 (25)	5 (18)	0.274	7 (25)	5 (18)	0.445	5 (18)	7 (25)	0.445	7 (25)	5 (18)	0.445
**Yes**	7 (25)	9 (32)		6 (21)	10 (36)		7 (25)	9 (32)		9 (32)	7 (25)		7 (25)	9 (32)	
**Frequency of changing bedsheet**															
**Weekly**	7 (25)	5 (18)	0.473 (F)	4 (14)	8 (29)	0.473 (F)	6 (21)	6 (21)	0.685 (F)	6 (21)	6 (21)	1.000 (F)	9 (32)	3 (11)	**0.043 (F)**
**2-Monthly**	2 (7)	2 (7)			2 (7)		2 (7)			1 (4)	3 (11)			2 (7)		2 (7)		2 (7)	2 (7)	
**Monthly**	4 (14)	8 (29)		7 (25)	5 (18)		7 (25)	5 (18)		6 (21)	6 (21)		3 (11)	9 (32)	
**Frequency of doing housekeeping**															
**Daily**	5 (18)	6 (21)	0.567 (F)	5 (18)	6 (21)	0.357 (F)	9 (32)	2 (7)	0.052 (F)	7 (25)	4 (14)	0.478 (F)	7 (25)	4 (14)	0.528 (F)
**Alternate day**	2 (7)	4 (14)			4 (14)		2 (7)			2 (7)	4 (14)			2 (7)		4 (14)		3 (11)	3 (11)	
**Weekly**	6 (21)	4 (14)			3 (11)		7 (25)			3 (11)	7 (25)			5 (18)		5 (18)		4 (14)	6 (21)	
**Monthly**	NIL	1 (4)		1 (4)	NIL		NIL	1 (4)		NIL	1 (4)		NIL	1 (4)	

**Table 4:** Association of each gene’s expression (*ZO-1*, *ZO-2*, *ZO-3*, *HDAC1* and *HDAC2*) with clinical parameters of AR patients (*n* = 28). *p* < 0.05 shown in bold.

**Characteristics**	***ZO-1*** **expression**			***ZO-2*** **expression**			***ZO-3*** **expression**			***HDAC1*** **expression**			***HDAC2*** **expression**		
	**<Median,** **n (%)**	**≥Median,** **n (%)**	***p*** **-value**	**<Median,** **n (%)**	**≥Median,** **n (%)**	***p*** **-value**	**<Median,** **n (%)**	**≥Median,** **n (%)**	***p*** **-value**	**<Median,** **n (%)**	**≥Median,** **n (%)**	***p*** **-value**	**<Median,** **n (%)**	**≥Median,** **n (%)**	***p*** **-value**
**Immediate family history of allergy**															
**No**	2	3	1.000 (F)	2	3	1.000 (F)	2	3	1.000 (F)	3 (11)	2 (7)	1.000 (F)	4 (14)	1 (4)	0.326 (F)
**Yes**	11	12		11	12		12	11		11 (39)	12 (43)		10 (36)	13 (46)	
**Classification**															
**Intermittent**	4 (14)	3 (11)	0.670 (F)	2 (7)	5 (18)	0.396 (F)	3 (11)	4 (14)	1.000 (F)	4 (14)	3 (11)	1.000 (F)	3 (11)	4 (14)	1.000 (F)
**Persistent**	9 (32)	12 (43)		11 (39)	10 (36)		11 (39)	10 (36)		10 (36)	11 (39)		11 (39)	10 (36)	
**Comorbidity (Conjunctivitis)**															
**No**	4 (14)	3 (11)	0.670 (F)	2 (7)	5 (18)	0.396 (F)	3 (11)	4 (14)	1.000 (F)	4 (14)	3 (11)	1.000 (F)	2 (7)	5 (18)	0.385 (F)
**Yes**	9 (32)	12 (43)		11 (39)	10 (36)		11 (39)	10 (36)		10 (36)	11 (39)		12 (43)	9 (32)	
**Comorbidity (Pharyngitis)**															
**No**	7 (25)	6 (21)	0.464	4 (14)	9 (32)	0.122	4 (14)	9 (32)	0.058	7 (25)	6 (21)	0.704 (F)	6 (21)	7 (25)	0.704(F)
**Yes**	6 (21)	9 (32)		9 (32)	6 (21)		10 (36)	5 (18)		7 (25)	8 (29)		8 (29)	7 (25)	
**Comorbidity (Sinusitis)**															
**No**	1 (4)	1 (4)	1.000 (F)	1 (4)	1 (4)	1.000 (F)	1 (4)	1 (4)	1.000 (F)	1	1	1.000 (F)	1	1	1.000 (F)
**Yes**	12 (43)	14 (50)		12 (43)	14 (50)		13 (46)	13 (46)		13	13		13	13	
**Comorbidity (Asthma)**															
**No**	10 (36)	7 (25)	0.102	6 (21)	11 (39)	0.142	8 (29)	9 (32)	0.699	9 (32)	8 (29)	0.699	10 (36)	7 (25)	0.246
**Yes**	3 (11)	8 (29)		7 (25)	4 (14)		6 (21)	5 (18)		5 (18)	6 (21)		4 (14)	7 (25)	
**Comorbidity (Otitis Media)**															
**No**	12 (43)	12 (43)	0.600 (F)	10 (36)	14 (50)	0.311 (F)	12 (43)	12 (43)	1.000 (F)	12 (43)	12 (43)	1.000 (F)	13 (46)	11 (39)	0.596 (F)
**Yes**	1 (4)	3 (11)		3 (11)	1 (4)		2 (7)	2 (7)		2 (7)	2 (7)		1 (4)	3 (11)	
**Comorbidity (Obstructive sleep apnea)**															
**No**	13 (46)	13 (46)	0.484 (F)	12 (43)	14 (40)	1.000 (F)	13 (46)	13 (46)	1.000 (F)	13 (46)	13 (46)	1.000 (F)	13 (46)	13 (46)	1.000 (F)
**Yes**	NIL	2 (7)		1 (4)	1 (4)		1 (4)	1 (4)		1 (4)	1 (4)		1 (4)	1 (4)	
**HDM (** ***D. farinae*** **)**															
**No**	3 (11)	2 (7)	0.639 (F)	3 (11)	2 (7)	0.639 (F)	2 (7)	3 (11)	1.000 (F)	3 (11)	2 (7)	1.000 (F)	5 (18)	NIL	**0.041 (F)**
**Yes**	10 (36)	13 (46)		10 (36)	13 (46)		12 (43)	11 (39)		11 (39)	12 (43)		9 (32)	14 (50)	
**HDM (** ***D. pteronysinnus*** **)**															
**No**	4 (14)	6 (21)	0.705 (F)	4 (14)	5 (18)	1.000 (F)	6 (21)	4 (14)	0.430	6 (21)	4 (14)	0.430	6 (21)	4 (14)	0.430
**Yes**	9 (32)	9 (32)		9 (32)	10 (36)		8 (29)	10 (36)		8 (29)	10 (36)		8 (29)	10 (36)	
**HDM (** ***B. tropicalis*** **)**															
**No**	6 (21)	3 (11)	0.228 (F)	3 (11)	6 (21)	0.435 (F)	4 (14)	5 (18)	1.000 (F)	4 (14)	5 (18)	1.000 (F)	7 (25)	2 (7)	0.103 (F)
**Yes**	7 (25)	12 (43)		10 (36)	9 (32)		10 (36)	9 (32)		10 (36)	9 (32)		7 (25)	12 (43)	

**Table 5:** Association of each gene’s expression (*ZO-1*, *ZO-2*, *ZO-3*, *HDAC1* and *HDAC2*) with demographical parameters of healthy controls (*n* = 28). *p* < 0.05 shown in bold.

**Characteristics**	***ZO-1*** **expression**			***ZO-2*** **expression**			***ZO-3*** **expression**			***HDAC1*** **expression**			***HDAC2*** **expression**		
	**<Median,** **n (%)**	**≥Median,** **n (%)**	***p*** **-value**	**<Median,** **n (%)**	**≥Median,** **n (%)**	***p*** **-value**	**<Median,** **n (%)**	**≥Median,** **n (%)**	***p*** **-value**	**<Median,** **n (%)**	**≥Median,** **n (%)**	***p*** **-value**	**<Median,** **n (%)**	**≥Median,** **n (%)**	***p*** **-value**
**Gender**															
**Male**	5 (18)	4 (14)	1.000 (F)	4 (14)	8 (29)	0.127	3 (11)	6 (21)	0.420 (F)	2 (7)	7 (25)	0.103 (F)	3 (11)	6 (21)	0.420 (F)
**Female**	9 (32)	10 (36)		10 (36)	6 (21)		11 (39)	8 (29)		12 (43)	7 (25)		11 (39)	8 (29)	
**Age (years)**															
**<28**	6 (21)	7 (25)	0.704	6 (21)	7 (25)	0.704	9 (32)	4 (14)	0.058	6 (21)	7 (25)	0.704	7 (25)	6 (21)	0.704
**≥28**	8 (29)	7 (25)		8 (29)	7 (25)		5 (18)	10 (36)		8 (29)	7 (25)		7 (25)	8 (29)	
**BMI**															
**<23.31**	7 (25)	7 (25)	1.000	9 (32)	5 (18)	0.131	7 (25)	7 (25)	1.000	6 (21)	8 (29)	0.450	7 (25)	7 (25)	1.000
**≥23.31**	7 (25)	7 (25)		5 (18)	9 (32)		7 (25)	7 (25)		8 (29)	6 (21)		7 (25)	7 (25)	
**Exposure to secondhand smoke**															
**No**	8 (29)	7 (25)	0.704	8 (29)	7 (25)	0.704	9 (32)	6 (21)	0.256	10 (36)	5 (18)	0.058	9 (32)	6 (21)	0.256
**Yes**	6 (21)	7 (25)		6 (21)	7 (25)		5 (18)	8 (29)		4 (14)	9 (32)		5 (18)	8 (29)	
**Home location**														
**Rural**	5 (18)	9 (32)	0.131	9 (32)	5 (18)	0.131	7 (25)	7 (25)	1.000	7 (25)	7 (25)	1.000	7 (25)	7 (25)	1.000
**Urban**	9 (32)	5 (18)		5 (18)	9 (32)		7 (25)	7 (25)		7 (25)	7 (25)		7 (25)	7 (25)	
**Living in an industrial area**															
**No**	14 (50)	11 (39)	0.222 (F)	12 (43)	13 (46)	1.000 (F)	12 (43)	13 (46)	1.000 (F)	13 (46)	12 (43)	1.000 (F)	14 (50)	11 (39)	0.222 (F)
**Yes**	NIL	3 (11)		2 (7)	1 (4)		2 (7)	1 (4)		1 (4)	2 (7)		NIL	3 (11)	
**Having pet**															
**No**	4 (14)	7 (25)	0.246	9 (32)	2 (7)	**0.007**	7 (25)	4 (14)	0.246	7 (25)	4 (14)	0.246	7 (25)	4 (14)	0.246
**Yes**	10 (36)	7 (25)		5 (18)	12 (43)		7 (25)	10 (36)		7 (25)	10 (36)		7 (25)	10 (36)	
**Frequency of changing bedsheet**															
**Weekly**	8 (29)	5 (18)	0.394 (F)	5 (18)	8 (29)	0.394 (F)	5 (18)	8 (29)	0.123 (F)	7 (25)	6 (21)	0.394 (F)	7 (25)	6 (21)	1.000 (F)
**2-Monthly**	NIL	2 (7)			2 (7)		NIL			NIL	2 (7)			2 (7)		NIL		1 (4)	1 (4)	
**Monthly**	6 (21)	7 (25)		7 (25)	6 (21)		9 (32)	4 (14)		5 (18)	8 (29)		6 (21)	7 (25)	
**Frequency of doing housekeeping**															
**Daily**	9 (32)	4 (14)	0.057 (F)	7 (25)	6 (21)	0.706 (F)	7 (25)	6 (21)	0.706 (F)	5 (18)	8 (29)	0.449 (F)	7 (25)	6 (21)	0.706 (F)
**Weekly**	4 (14)	10 (36)			6 (21)		8 (29)			6 (21)	8 (29)			8 (29)		6 (21)		6 (21)	8 (29)	
**Monthly**	1 (4)	NIL		1 (4)	NIL		1 (4)	NIL		1 (4)	NIL		1 (4)	NIL	

*HDAC2* expression did not differ significantly between healthy controls and AR patients in terms of one or multiple types of HDMs sensitization (Fig. S1A) using Kruskal-Wallis test. Dunn’s multiple comparison test showed no significance between each group (Fig. S1A). Other test on *HDAC2* expression of healthy controls and AR patients compared to frequency of changing bedsheets also showed no significance between each group (Fig. S1B).

## Discussion

AR is a chronic and prevalent nasal mucosa inflammatory disorder, with the pathophysiologic mechanisms responsible for the severity of disease being only partially understood (Wang et al., 2020a; Wang et al., 2020b). Nasal epithelial barrier defects in AR patients have been hypothesized to result in excessive allergen exposure and activation of inflammatory cells (Steelant et al., 2018). In this context, the role of the nasal epithelium in protecting the nasal airway from allergens exposure is still not clearly understood.

In this study, we demonstrated that decreased expression of ZO-1 occurred in HDM-sensitized moderate-severe AR patients compared to healthy controls. This was in line with findings of previous studies in AR patients that showed impaired epithelial TJ function and lower ZO-1 mRNA expression in both nasal epithelial cells and nasal biopsy specimens (Steelant et al., 2016; Wang et al., 2020a; Wang et al., 2020b). Independent studies also showed significantly decreased expression of ZO-1 protein in nasal epithelium of AR patients compared with healthy controls (Wang et al., 2020a; Wang et al., 2020b). Immunofluorescence staining demonstrated a relatively poor ZO-1 arrangement in biopsy specimens of HDM-induced AR patients compared to healthy controls (Steelant et al., 2016). HDMs are known to contain abundant proteases (Reithofer & Jahn-Schmid, 2017), and protease activity of HDMs increases the permeability of epithelial barrier that promotes the penetration of allergens into nasal mucosa by targeting TJs (Wan et al., 1999; Roche et al., 2000; Okano, 2009). Reduced expression of ZO-1 was also observed in patients with CRS with nasal polyps, asthmatic patients, eosinophilic esophagitis (EoE) and these impaired epithelial TJ function are considered to be part of the pathophysiology (De Benedetto et al., 2011; Salim & Söderholm, 2011; Xiao et al., 2011; Soyka et al., 2012; Katzka et al., 2014). The airway epithelium is known as the first line of defense that acts as a protective barrier against environmental allergens (Nadeem et al., 2019). Impaired expression of ZO-1 may thus facilitate the infiltration of allergens into the epithelial barrier of AR patients.

In contrast to ZO-1, expression levels of ZO-2 and ZO-3 did not significantly differ between AR patients and controls in our study. Recent study demonstrated that lower expression of ZO-2 and ZO-3 were observed in nasal biopsy samples of atopic patients in Turkey (Yılmaz et al., 2019). Epidemiological studies showed that cigarette smoke is one of a risk factor for asthma and CRS with polyps. Incubation of 16HBE14 cells and human bronchial epithelial cells (pHBECs) with cigarette smoke extract decreased ZO-2 expression and thus disrupted TJ barrier (Fukuoka & Yoshimoto, 2018). However, there are still no studies investigating the expression of ZO-2 and ZO-3 molecules in AR patients.

MAGUKs (ZO-1, ZO-2 and ZO-3) are thought to play important roles in producing and maintaining specialized membrane domains in various types of cells (Inoko et al., 2003). Similar to ZO-1, both ZO-2 and ZO-3 are localized at TJs in epithelial cells and bind directly to the actin filaments (Itoh et al., 1997; Fanning et al., 1998). Nevertheless, the contribution of both ZO-2 and ZO-3 to the nasal epithelial barrier remains unclear due to lack of studies on the involvement of both ZO-2 and ZO-3 in AR pathogenesis.

Pertaining to HDACs, we did not observe significant difference in the expression levels of HDAC1 and HDAC2 in AR patients compared to controls. HDAC1 can suppress TWIK-related potassium channel-1 (Trek1) in the nasal mucosa essential in preserving the epithelial barrier function (Bittner et al., 2013; Jiang et al., 2015), establishing HDAC1 as one of the key players in deregulating epithelial barrier. Recent therapeutic research has demonstrated that HDACi enhance ZO proteins expression, leading to improved epithelial barrier integrity (Yılmaz et al., 2019). HDACi such as NaB and JNJ-26481585 inhibit the activity of HDAC1 resulting in enhanced nasal epithelial barrier integrity (Steelant et al., 2019; Wang et al., 2020a; Wang et al., 2020b). Furthermore, treatment of fetal human lens epithelial cells with the HDACi TSA increased the expression of ZO-1 and improved the epithelial barrier integrity (Ganatra et al., 2018). Previous study reported higher levels of HDAC1 in AR patients compared to healthy controls in China (Wang et al., 2015). This may be due to prevalence of seasonal allergens in China (*e.g.,* pollens) (Zhang et al., 2009) but distinct from perennial allergens (*e.g.,* HDMs) in Malaysia.

Interestingly, we observed that higher expression of HDAC2 in AR patients was significantly associated with lower frequency of changing bedsheet. HDMs mainly come into contact with human through mattresses and bedsheet (Abu Khweek et al., 2020). A study in China also showed that high concentration of *D. farinae* was detected on rhinitis children’s beddings compared to controls (Huang et al., 2019). Hypersensitivity to HDMs also contribute to atopic sensitization in 50–85% of asthmatics, and are strong inducers of causing allergy in worldwide population (Abu Khweek et al., 2020). HDAC activity has been identified as a key factor of allergic inflammation and TJ dysfunction (Steelant et al., 2019). Increased production of Th2 cytokines caused by allergic inflammation leads to increased HDAC activities in epithelial cells that augment mucosal permeability (Steelant et al., 2018). This is in line with higher expression of HDAC2 which was significantly associated with our cohort of AR patients sensitized to *D. farinae*. Collectively, this suggests that HDACi administration is a potential therapeutic strategy for AR patients sensitized to *D. farinae* and this is subject to future investigations.

In this study, we observed that the expression of ZO-2 was higher in healthy controls who had pets (Table 5). Exposure to pets at early stage may decrease the possibility of developing allergic sensitization to animal dander or other inhaled allergens (Shargorodsky et al., 2017). It has been shown that living with cats and dogs was associated with lower risk of developing atopy during childhood and young adulthood (Päivi & Darryl, 2009). Frequent exposure to allergens may later protect from sensitization by adapting to transient increase of Th2 cytokines levels and subclinical responses (Shargorodsky et al., 2017; Steelant et al., 2018). However the role of Th1 is still not clearly understood (Steelant et al., 2018).

We acknowledge the limitations of the study as follows: (1) We focused on HDM-sensitized AR patients only without the inclusion of other allergens during SPT. It is plausible that our cohort of AR patients were also allergic to other allergens which may contribute to the TJs disruption; (2) In our study, the gene expression was sufficient to demonstrate the presence of ZO and HDAC. Although protein expression data is important to confirm the existence of ZO and HDAC gene, however we could not perform the protein analysis in this study due to budget constraint. (3) We did not measure specific IgE tests for all the allergens. In the future, we would like to further investigate more on protein expression levels, which will help to strengthen our research.

## Conclusions

In summary, our data indicate that ZO-1 is a key TJ molecule whose reduced expression is associated with defective nasal epithelial barrier integrity in HDM-sensitized AR patients. These results support development of therapies that restore ZO-1 expression in nasal epithelial cells of AR patients. The expression profile of ZO-2, ZO-3 and HDAC2 were demonstrated in AR patients relative to non-allergic subjects for the first time. Collectively, dysregulated ZO-1 and HDAC2 expression levels may play key roles in the onset of AR through disruption of nasal epithelial barrier integrity.

## Supplemental Information

10.7717/peerj.13314/supp-1 Figure S1 Relative *HDAC2* expression (A) Relative *HDAC2* expression in healthy controls (HC) and AR patients sensitized with any HDM allergen (AR-1-HDM), AR patients sensitized with two HDM allergens (AR-2-HDMs) and AR patients sensitized with three HDM allergens (AR-3-HDMs). (B) Relative *HDAC2* expression in HCs and AR patients with their frequency of changing bedsheet of monthly (AR-monthly) and two-monthly (AR-2-Monthly). NS: Not significant.

10.7717/peerj.13314/supp-2 File S1 Calculation for 2^−ΔΔCt^ value of ZO-1, ZO-2, ZO-3, HDAC1 and HDAC2

## Additional Information and Declarations

### Data Availability

The following information was supplied regarding data availability: Raw data are available in the Supplemental Files.
