# Supplementary figures and images for: Zonula occludens-1 expression is reduced in nasal epithelial cells of allergic rhinitis patients
Source: PeerJ. 2022 Apr 22;10:e13314. doi: 10.7717/peerj.13314 (PMC9037125; doi:10.7717/peerj.13314)

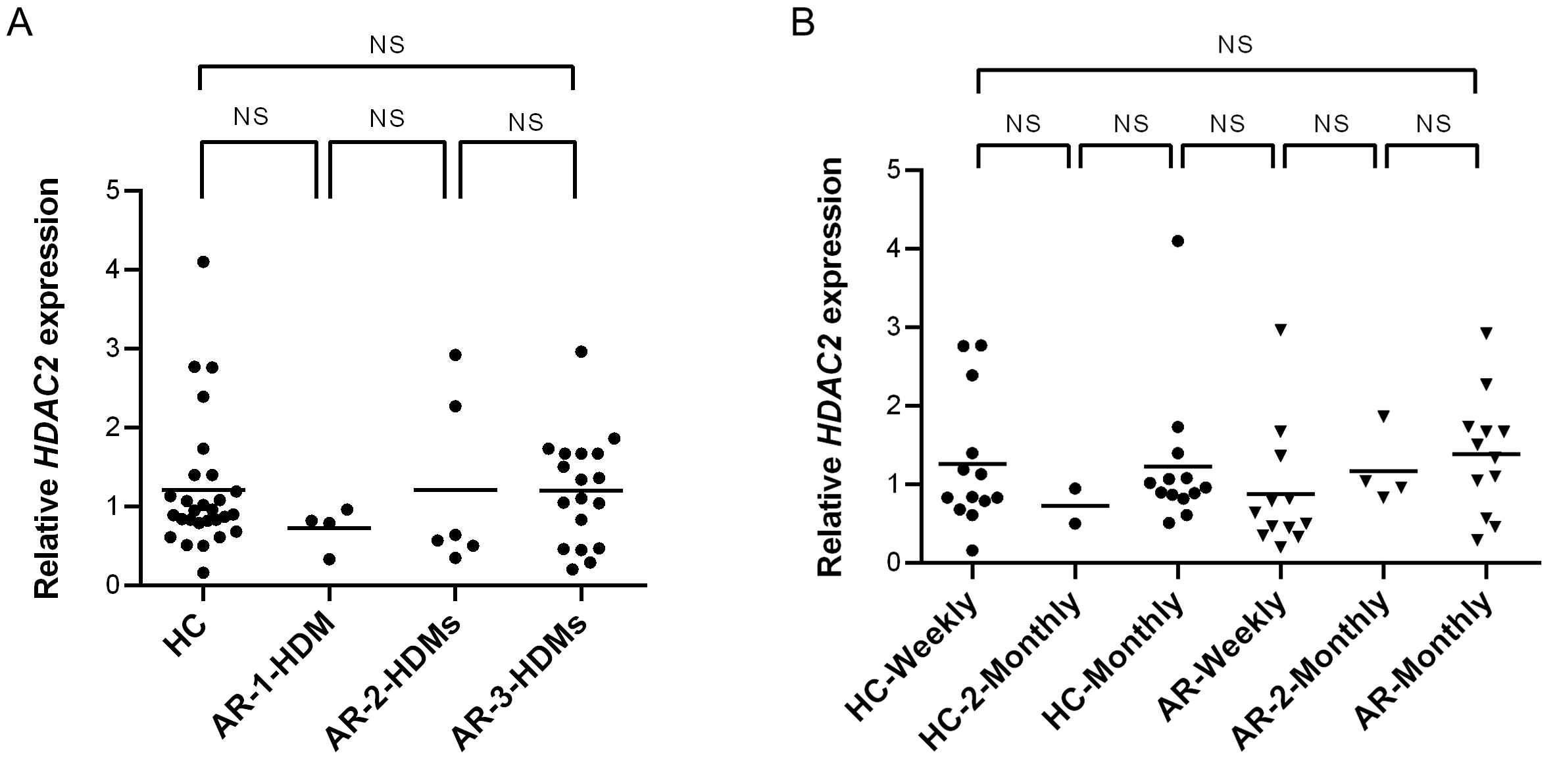

Supplement: Figure S1 — (A) Relative HDAC2 expression in healthy controls (HC) and AR patients sensitized with any HDM allergen (AR-1-HDM), AR patients sensitized with two HDM allergens (AR-2-HDMs) and AR patients sensitized with three HDM allergens (AR-3-HDMs). (B) Relative HDAC2 expression in HCs and AR patients with their frequency of changing bedsheet of monthly (AR-monthly) and two-monthly (AR-2-Monthly). NS: Not significant. [file peerj-10-13314-s001.jpg]
